# Energy Use of Flux Salt Recovery Using Bipolar Membrane Electrodialysis for a CO_2_ Mineralisation Process

**DOI:** 10.3390/e21040395

**Published:** 2019-04-12

**Authors:** Evelina Koivisto, Ron Zevenhoven

**Affiliations:** Thermal and Flow Engineering Laboratory, Åbo Akademi University, Piispankatu 8, 20500 Turku, Finland

**Keywords:** bipolar membrane electrodialysis, ammonium sulfate, ammonium bisulfate, CO_2_ sequestration, mineral carbonation

## Abstract

Mineral carbonation routes have been extensively studied for almost two decades at Åbo Akademi University, focusing on the extraction of magnesium from magnesium silicates using ammonium sulfate (AS) and/or ammonium bisulfate (ABS) flux salt followed by carbonation. There is, however, a need for proper recovery and recirculation of chemicals involved. This study focused on the separation of AS, ABS and aqueous ammonia using different setups of bipolar membrane electrodialysis using both synthetic and rock-derived solutions. Bipolar membranes offer the possibility to split water, which in turn makes it possible to regenerate chemicals like acids and bases needed in mineral carbonation without excess gas formation. Tests were run in batch, continuous, and recirculating mode, and exergy (electricity) input during the tests was calculated. The results show that separation of ions was achieved, even if the solutions obtained were still too weak for use in the downstream process to control pH. Energy demand for separating 1 kg of NH_4_^+^ varied in the range 1.7, 3.4, 302 and 340 MJ/kg NH_4_^+^, depending on setup chosen. More work must hence be done in order to make the separation more efficient, such as narrowing the cell width.

## 1. Introduction

**Figure 1:**
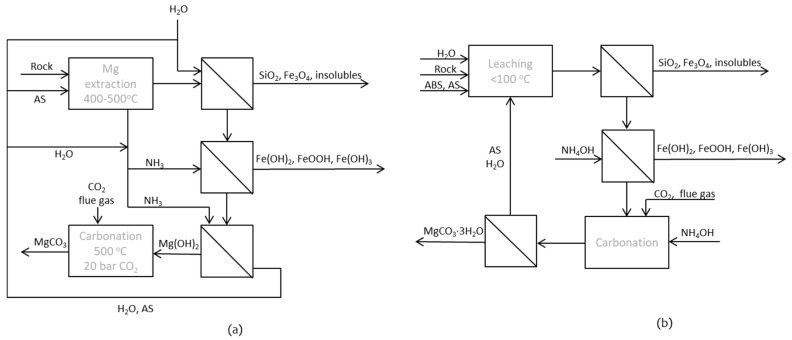
(**a**) Conventional dry-wet-dry ÅA route; (**b**) wet ÅA route.

The total greenhouse gas emissions in Finland in 2017 were 15.8 million tonnes CO_2_ eq. (approximately 22%) less than in 1990, according to preliminary data for 2017 from Statistics Finland [1]. The target for 2050 is that the greenhouse gas emissions will be reduced by at least 80% compared to the emission levels in 1990 [2,3]. One way to reduce CO_2_ emissions is to find sustainable options for CO_2_ storage or sequestration. Finland is lacking the possibility to store CO_2_ underground, which makes it important to find alternative options. Mineral carbonation routes have therefore been extensively studied for almost two decades at Åbo Akademi University (ÅA) [4,5,6,7,8]. The process developed at ÅA, the ÅA route, involves letting magnesium silicate rock (preferably containing serpentinite, Mg_3_Si_2_O_5_(OH)_4_) react with ammonium sulfate (AS) to extract magnesium, which subsequently reacts with CO_2_ of flue gas and form stable magnesium carbonates. A challenging step in the ÅA route is the extraction of magnesium from the serpentinite rock [4,6,8]. Ammonium bisulfate (ABS) has also been used as input chemical and one option is a pH-swing process sometimes referred to as the UK route [9,10]. As a combination of the two routes, an option could be to use both AS and ABS for aqueous leaching of magnesium from the rock [11] or replace AS with ABS in the ÅA route [12]. However, proper recovery and recirculation of the chemicals involved need to be optimized independent of the chemical being used. Different options for optimizing the process have recently been presented [13]. The possibility of separating ABS from the process stream using alcohols has been studied by others, a method that in turn would require the regeneration of the alcohol [14]. A comparison between the aqueous mineral process route involving leaching and a wet carbonation step, and the process route involving a dry extraction step at elevated temperature (400–500 °C) followed by dissolution and separation of insoluble matters and finally a dry carbonation step was given in [10]. The process routes are schematically shown in Figure 1.

**Figure 2:**
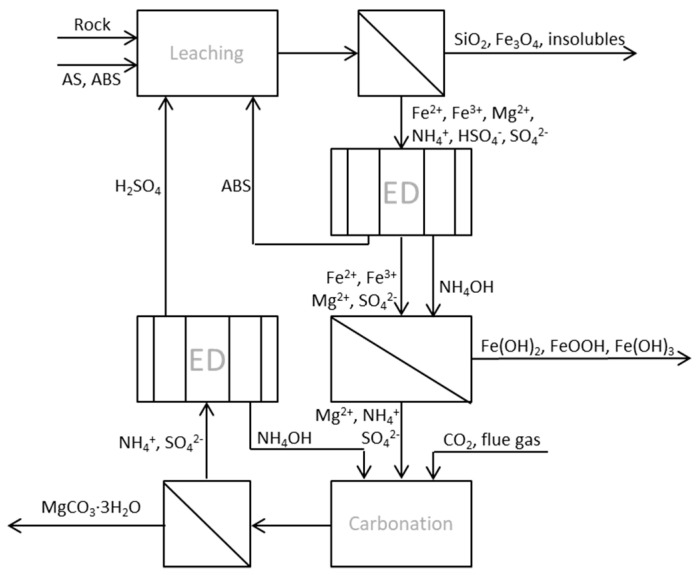
Process scheme of ÅA route 3 with two integrated ED units for chemical regeneration.

Evaporation of water or crystallization of AS for the dry extraction step is needed in the conventional ÅA route, which is also quite energy demanding. In cases where the wet process route is chosen, it should be possible not only to stepwise regenerate ABS and AS, but also the ammonia needed for ion precipitation and carbonation by using bipolar membrane electrodialysis (BPMED). There have been few studies on the separation of ammonium bisulfate from ammonium sulfate or ammonia by electrodialysis (ED). The regeneration of sulfuric acid and ammonia from ammonium sulfate in waste waters from acid production was studied [15], resulting in an electricity consumption of 0.207 kWh/mol sulfuric acid, which corresponded to a current density of 30 mA/cm^2^ and average current efficiency (ACE) of 68.7%. The latter can, in short, be explained as the ratio between the amount of sulfuric acid multiplied by 100 and the theoretical concentration that may be obtained according to Faraday’s law. A somewhat similar approach was used as part of this study, aiming at either (1) separating ammonium bisulfate from the process stream immediately after filtering the insoluble matters left after the leaching step, or (2) regenerating ammonia and sulfuric acid from the solution that remains after carbonation. The alternative process route (ÅA route 3) with applied ED is described in Figure 2, with all process steps occurring in aqueous solution without dry (thermal) extraction and dry carbonation involved.

Modern separation methods based on membrane technology can contribute to the increased energy efficiency of processes that fix carbon dioxide into solid carbonate materials. This combines increased sustainability of resources use with reduced greenhouse gas emissions. This article presents work done in order to separate and produce AS, ABS and aqueous ammonia, respectively, by different setups of a bipolar membrane electro dialysis (BPMED) setup. Initial work using monovalent cationic and monovalent anionic membranes in order to separate ABS from AS was reported recently [16]. This study is a continuation of that work. Case studies have been made in cooperation with industry partners who, unfortunately, have not so far recognized the economic viability of using one of the ÅA routes, operating on pre-separated CO_2_ [6,17], or preferably flue gas, directly [18,19], see also [10,20,21].

### Bipolar Membranes (BPM)

**Figure 3:**
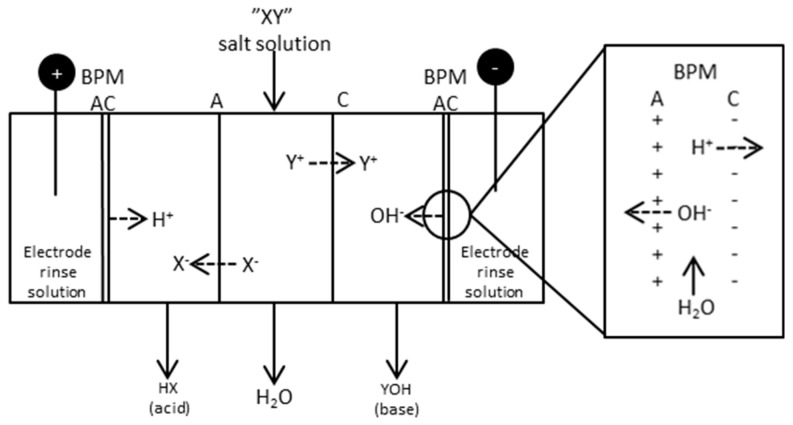
Principle scheme of a five compartment BPMED cell. The square to the right shows the principle of a bipolar membrane, A = monovalent anion membrane; C = monovalent cation membrane.

Bipolar membrane separations have attracted interest in recent years because of the many possible application areas, e.g., in pollution control and chemical processing [22]. The preparation of BPM material was already described in 1956 [23]. BPMs are layered membranes consisting of both a cationic and anionic side with a thin water film separating the membranes. This makes it possible to split water into its ions, i.e., H^+^ and OH^−^, thus making it possible to produce acid and bases from its salt. Figure 3 shows a sketch of a five compartment BPMED cell where a salt “XY” is fed to one of the compartments, resulting in an acid “HX” and a base “YOH”.

The advantages of BPMs are many and include, especially for this study, the possibility of regenerating and reusing input chemicals. Gas formation is also avoided by using BPMs because of the electrochemical reactions linked to the splitting of water molecules. The high potential of BPMs in different applications is one reason they have gained increased attention in recent decades. Thanks to the acid-base splitting effect it is possible to apply BPMs in a way so that combined reaction and purification processes are obtained [24,25], which is also the case in this study.

Several physical and chemical factors will affect the efficiency and electricity consumption of the stack. The low transport rate of co-ions (ions with similar charge), good thermal and chemical stability in both strong acids and bases, together with a high water dissociation rate in addition to low electrical resistance at high current density are some important factors. The electricity consumption is also linked to the conductivity of the anionic and cationic membranes used with the bipolar membranes in an electrodialysis stack, together with the characteristics of the BPM. Temperature also affects the electricity consumption and will increase the water splitting rate while decreasing the electrical resistance of the electrolytes as temperature increases [26].

The BPMs will be used here in different setups to see if separation by BPMED could act as a possible option for the regeneration of the chemicals involved in the ÅA route. The focus in this study will be the possibility of separating acids and bases, but also the energy demands of those separations. An energy efficient regeneration unit is crucial for being able to implement BPMED to the ÅA route. The energy need for recovery of a specific amount of chemical will give an indication of how efficient the separation will be using the setups assessed here.

## 2. Materials and Methods

Different setups of a BPMED stack with one repeating unit were tested with the aim of separating either AS, ABS or ammonia from the process stream. Both synthetic solutions and rock-derived solutions were used as feed stock. Three different Eurodia membranes were combined [27]: monovalent anion membranes (ACS), monovalent cation membranes (CMS) and bipolar membranes, respectively. The combinations are described in Section 2.2 and Section 2.3, below. The ED stack was designed either in the way that it would be placed immediately after leaching and filtering of the leached rock (Section 2.2), or after the carbonation step (Section 2.3), where ideally only ammonium sulfate and some excess ammonia should be left in the solution. The results will be presented separately under Section 3.

**Figure 4:**
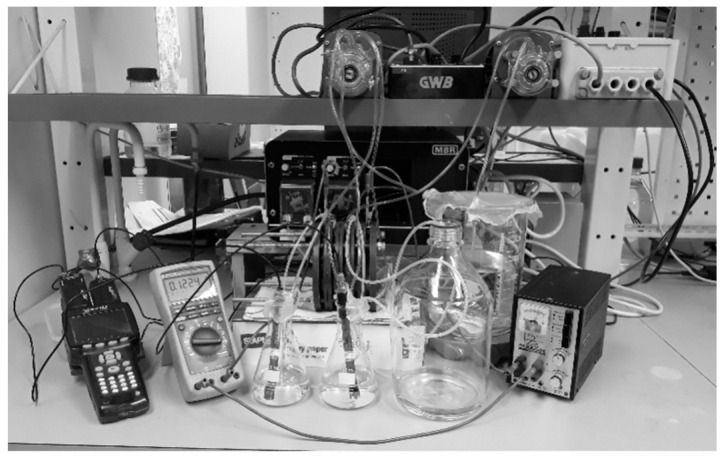
ED setup with pH meter and multimeter (**left**), power supply (**right**), vessels and ED stack (**middle**) and pumps (**top**).

Synthetic solutions were prepared with different amounts of ammonium sulfate (Merck, 99.5%), ammonium bisulfate (Acros organics, 99%), aqueous ammonia (25% in water), magnesium sulfate (VWR Chemicals, 99.6%, iron(III) sulfate hydrate (VWR Chemicals, Fe(II) max 0.05%) and iron(II) sulfate heptahydrate (VWR Chemicals, Fe(III) max 0.02%). The rock-derived solutions were obtained by letting serpentinite siderock from the Hitura nickel mine in Finland react with 0.8 L of a 0.7 M AS + 0.7 M ABS solution for 3 h at 70 °C. The particle size of the rock was 63–125 µm. The insoluble matter was filtered and the solution was tested at different parameters and different setups in the ED stack (Figure 4).

The ED stack consisted of 18 mm wide compartments, with a membrane area of 77 cm^2^ and a volume of 139 cm^2^. Ti/Pt anode and cathode electrodes (Ti-shop, London, UK) in the end compartments. The BPMs were placed at the end of the stack in all setups, and the BPMED was run at initial voltages of either 20 V or 30 V.

The tests were run (1) continuously, with an inlet and an outlet at the respective compartments; (2) with recirculation of all or some of the compartment solutions; or (3) batch-wise. Measurements of pH and conductivity were done either directly in the solution (continuous/recirculation mode), or by taking samples of 10 mL from the cell (batch mode). Each compartment was filled with 135 mL solution/H_2_O at the beginning of a batch test.

Several end solution samples were sent to Eurofins Scientific (Lahti, Finland) for SO_4_^2−^, NH_4_^+^, Fe, Mg and Ni analyses. Only the rock-derived feedstock was analyzed with respect to nickel and the presence of nickel ion in the concentrate outlet. Ni ion being either I+ or II+ charged makes it possible that at least Ni(I) ions will pass over to other compartments, which must be controlled, for example in cases where nickel-containing serpentinite is used, like the Hitura siderock that was used here. The Hitura rock sample composition is given in Table 1.

### 2.1. Exergy Calculations and Calculations of the Energy Need for Separating 1 kg of Ammonium

The input energy, i.e., electric power, is equal to the input exergy. Exergy is extensively described in [28]. By knowing the electric current (*I*, C/s) the voltage (*U*, J/C) and the time (*t*, s), it is possible to calculate the input exergy in joules after time *t* as

(1) $$Ex_{in}\left(t\right)=P_{in}=I\cdot U\cdot t$$

and the exergy input (*Ex_in_*) for a time interval (*t*_1_, *t*_2_) was then calculated from the electric power input as average values for corrected voltages as;

(2) $$Ex_{in}=\frac{U_{1}+U_{2}}{2}\cdot \frac{I_{1}+I_{2}}{2}\cdot \left(t_{2}-t_{1}\right)$$

where *U* is the voltage (V), I is the electric current (A) and *t* is time (s). The length of the time intervals varied with experimental conditions from 15 to 30 min and is also the time between two subsequent sampling times. Equation (2) gives an estimation of the exergy input. Most of the time, either voltage or electric current was constant. The calculated exergy for intervals in a test was summed to a total cumulative exergy input at any time and is presented as figures in the results section. This way of calculating input exergy was also used in [16].

The energy needs for separating 1 kg of NH_4_^+^ from the process stream were also calculated for four different test approaches in this study and compared between each other. The calculations were either based on the decrease of ammonia in the synthetic solution measuring ammonium concentration by ISE electrodes, or based on the external ammonium analyses in the basic solutions obtained. pH in the basic solutions exceeded pH 6.5 by far, which means that the majority of ammonium was found as dissolved molecular NH_3_ in basic solutions with high pH. Below pH 6.5, 100% was present as NH_4_^+^, while at pH 10, 95% was present as NH_3_, increasing to 100% NH_3_ at pH 11.5. This was also taken into account by using the equilibrium constant for ammonium, K_b_ = 1.8 × 10^−5^ and the reaction of ammonium and ammonia in Reaction (3):

NH_3_ + H_2_O ⇌ NH_4_^+^ + OH^−^ (3)

combined with Expression (4) for K_b_

K_b_ = [NH_4_^+^]·[OH^−^]/[NH_3_] (4)

### 2.2. BPMED after Leaching Step

**Figure 5:**
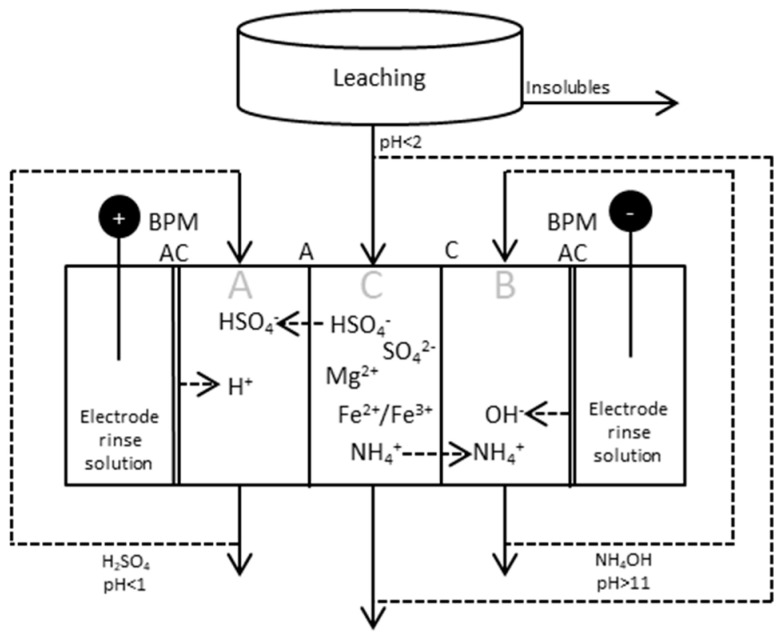
BPMED setup for production and separation of sulfuric acid and ammonia from the process stream after the leaching step. The process can be run either in batch mode, with recirculation of all/some of the three different streams, or continuously; i.e., running the process with inlets and outlets for the respective compartments.

The principle scheme of ED applied after leaching and filtering is given in Figure 5, together with test parameters and the compositions of the starting solutions in Table 2 and Table 3, respectively.

The leachate was at the beginning of the tests fed to the middle compartment, while distilled water was fed to the acidic and alkaline compartments, respectively. Samples of 10 mL were taken from the cell every 15 min and analyzed with respect to pH, NH_4_^+^ and conductivity. NH_4_^+^ was measured using Nico 2000 ion selective electrodes (ISE) in the tests using low concentrations of AS and ABS (0.05 M) in the starting solution, i.e., tests (3)–(5). Samples from test (11) and tests (13)–(15) were sent to Eurofins Scientific for analysis.

An ACS and a CMS membrane were put together in two initial tests to see if they could behave as a BPM. As expected, this did not work, since the monovalent membranes used do not transport water molecules, and the BPMs require a thin water film between the membrane layers to be able to split water into ions. Voltages of 20 and 30 V, respectively, were applied, but almost no electric current could go through the stack. These tests are referred to as test (1) and (2) in Table 2 and in Table 3. Results from these tests will not be presented any further.

### 2.3. BPMED after Carbonation Step

**Figure 6:**
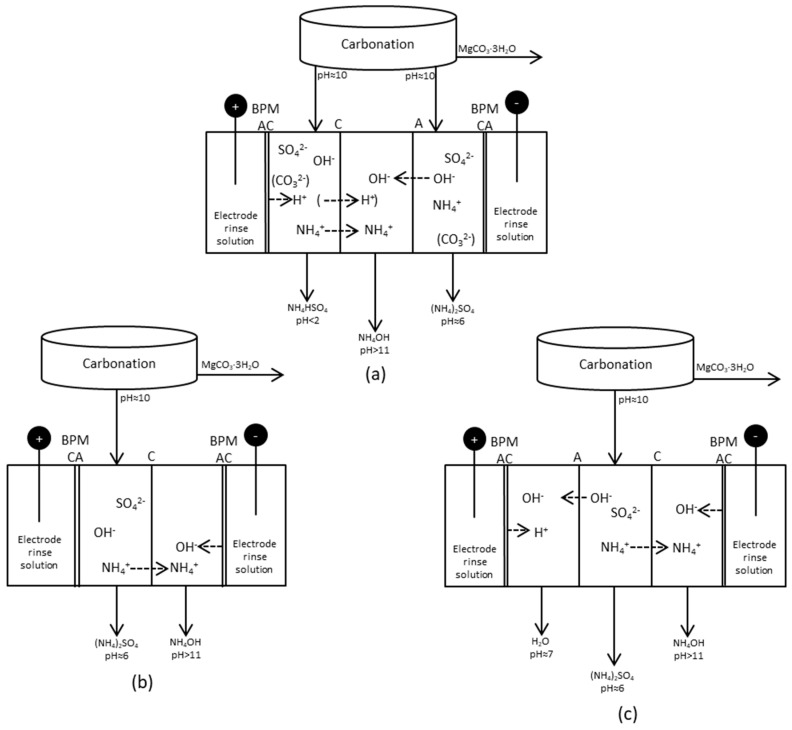
Different setups for production and separation of species after the carbonation step. Setup (**a**): Five compartment cell, ammonia collected in the middle compartment; Setup (**b**): Four compartment cell, ammonium ions collected and combined with OH^−^ from a BPM; Setup (**c**): Five compartment cell, ammonium collected and combined with OH^−^ from a BPM at the same time as OH^−^ are removed from the process solution and combined with H^+^ from another BPM.

Synthetic solutions were prepared for BPMED tests with the aim to regenerate and separate AS and/or ABS and ammonia after the carbonation step. Four tests in total were run in batch mode with three different setups of the BPMED stack. All tests were run at an initial 30 V. The electrode rinse solution was 0.15 M AS, and the solution was recirculated between the anode and cathode compartments during the test. Total test time was 135 min for all tests. 1.0 M or 0.15 M AS solutions were prepared before all tests, and a 25% ammonia solution was thereafter added in order to increase pH to 10, which is the pH that should be maintained at the carbonation step. Figure 6 and Table 4 shows the process parameters for these tests. pH and conductivity were measured by taking 10 mL samples every 15 min from the compartment where ammonia was collected.

## 3. Results and Discussion

The results will be presented in two sections. Section 3.1 reports on the tests done at conditions after the leaching step using either synthetic or rock-derived solutions, followed by reports on tests done at conditions after the carbonation step in Section 3.2.

### 3.1. BPMED after Leaching

All tests in this section were done using the setup shown in Figure 5. Process parameters and composition of starting solution are given in Table 2 and Table 3.

#### 3.1.1. Initial BPMED Batch Mode Tests (Tests (3)–(5))

**Figure 7:**
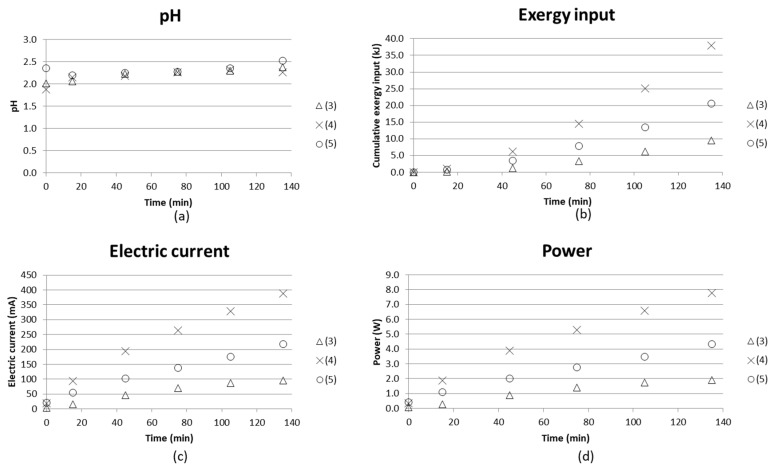
(**a**) pH in concentrate compartment; (**b**) cumulative exergy input; (**c**) electric current and (**d**) power as function of time for initial tests with BPMED setup for separation of acid and base from (3) synthetic solutions of AS and ABS with water in electrode compartments; (4) replaced with AS and ABS solution in electrode compartments; and (5) adding iron (II) sulfate and magnesium sulfate to the concentrate.

pH, cumulative exergy input, electric current and power as function of time are given in Figure 7. The diagrams compare batch mode tests with synthetic starting solutions of AS and ABS in the middle compartments and distilled water in the nearby compartments, together with

(3) water in the electrode compartments;

(4) water replaced with AS+ABS solution in the electrode compartments; and

(5) keeping AS+ABS in the electrode compartments and adding iron(II) sulfate and magnesium sulfate to the synthetic solution.

The cumulative exergy consumption as presented in Section 2.1 was calculated in the same manner as in the previous study [16], where electric energy could be directly calculated as exergy input.

Hardly any difference in pH in the concentrate compartment could be noticed from these three tests, while the exergy input becomes higher as AS+ABS is used in the electrode compartments ((4) and (5)). This could be explained by the fact that the electrode compartments will have more ions, which can contribute to the current going through the stack.

However, in the case where iron and magnesium sulfate were added to the starting solutions (5), the current was found to be a bit lower than using only AS+ABS solution (4). One reason for this could be that magnesium forms complexes with hydroxides [29,30,31] which would affect the current going through the cell since the hydroxide complexes could cause fouling of the membranes. The membranes used can be examined, e.g., by microscopy methods in order to see if fouling did occur. This is planned as future work.

**Figure 8:**
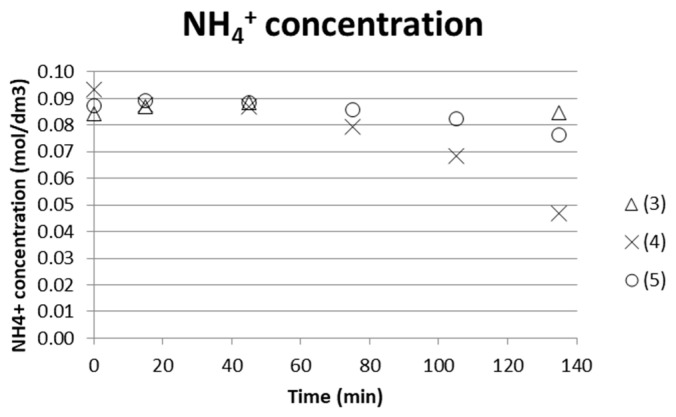
NH_4_^+^ for three tests run with low initial concentrate AS and ABS (0.05 M, respectively) solutions measured by ammonium ISE.

ISE electrodes were used to analyze the separation degree of ammonia from the middle compartment versus time. The 10 mL samples taken were fed back to the stack after analysis, which makes the concentration indicative rather than quantitative on the degree of separation. Figure 8 shows that the test using only AS+ABS starting solution together with a similar solution composition in the electrode compartments gives better separation. The (measured) concentration decreased from approx. 0.09 M to 0.045 M, which implies a separation degree of 50%.

The energy requirement for separating 1 kg NH_3_/NH_4_OH was estimated by comparing the [NH_4_^+^] separated from the process stream and with the input exergy at the end of the test. Taking test (4) as an example gave 0.045 M NH_4_^+^ separated (Figure 8), and cumulative input exergy 38 kJ after a time of 135 min (Figure 7) → 0.045M NH_4_^+^ in a 135 mL solution with molar mass M ≈ 18 g/mol gave m(NH_4_^+^) = 0.10935 × 10^−3^ kg/38 kJ. This corresponds to a value of 350 MJ/kg NH_4_^+^ separated. ED stacks with tens or even hundreds of membranes in a sequence are common practice in larger scale, which will increase the efficiency of the process. The compartment width used here was 18 mm, which enabled measuring and building a laboratory scale setup for initial tests. However, it is important to keep the compartment width as small as possible; even below 1 mm is common. The smaller the compartment width, the less energy is consumed, since the chemical resistance in the solution is high. Tests using an ED setup with more repeating units and narrower compartment width are therefore needed in order to obtain more reasonable energy demands for separation. Much of the energy will in these tests with only one repeating unit go to water electrolysis at the electrodes.

#### 3.1.2. Interruption and Recirculation Instead of Continuous Flow (Tests (6)–(8))

**Figure 9:**
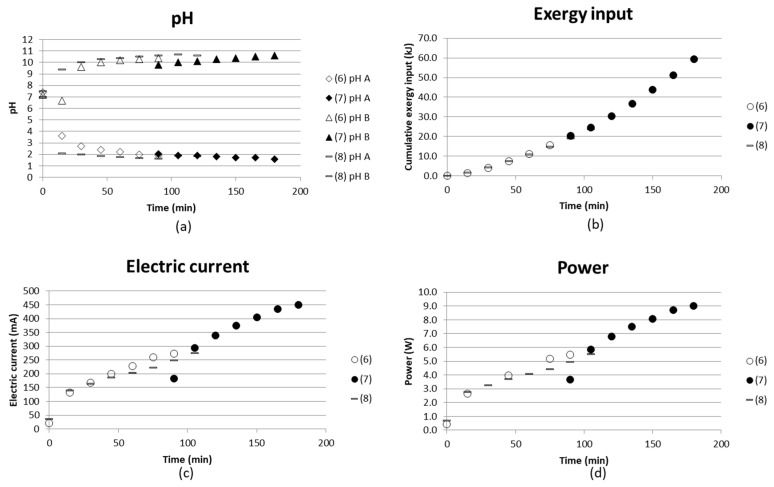
(**a**) pH in acidic and basic solution; (**b**) cumulative exergy input; (**c**) electric current in mA; and (**d**) power (W) as a function of time for two similar tests run after each other without emptying the stack in between ((6), (7). (8) is a test with a continuous flow instead of recirculation in the concentrate compartment.

Test (6) and (7) were performed after each other, i.e., test (6) was run at 20 V for 90 min, after which the voltage was switched off. The solutions were left in the stack and the test was continued on the second day with test (7), when a voltage of 20 V was applied for 90 min again. All solutions were recirculated back to their individual compartment with a flow rate of 1 L/h and a total volume of each solution of 500 mL. Figure 9 shows how pH changed in the solution left in the stack overnight, and how it continues from the first test levels to change when the voltage is applied again. This simple test shows that there was a change in the pH of the basic solution, while the pH of the acidic solution remained almost unchanged overnight. The decrease of the pH in the basic solution could be explained by the evaporation of ammonia. The current already returned to the same levels at the end of test (6) after the first measurement time of 15 min.

No analyses of ammonium ions were made for these tests, but they gave an indication of the state in the stack if the voltage was switched off and the test was later continued.

Test (8) was similar to tests (6) and (7), but the concentrate solution was run as a continuous flow, i.e., the outlet was collected and the pH measured in the outlet solution. No remarkable difference was seen when comparing tests (6) and (8) with each other. Larger volumes could probably have resulted in larger differences. The measuring took place in the recirculated solution or in the collected outlet. Measuring pH only in a smaller volume before entering the outlet vessel could also give different results.

#### 3.1.3. Continuous Flow Tests at 30 V (Tests (9)–(12))

All tests in this section were run in continuous mode in all compartments with varying inlet flow rates at 30 V. The concentrations were also higher for these tests, and were derived from a test done by [11], leaching magnesium from 10 g of Hitura serpentinite rock with particle size 62–125 µm in 0.4 L of 0.7 M AS+0.7 M ABS solution at 70 °C for 3 h. The analyses from this test served as template for the composition of the starting solution in tests (9) to (12). The initial concentration of AS was assumed to remain at 0.7 M, while ABS is 0.54 M. The reason for this is given by following reactions:

Mg_3_Si_2_O_5_∙(OH)_4_ (s) + 3(NH_4_)_2_SO_4_(aq)→ 3MgSO_4_(aq) + 2SiO_2_(s) + 6NH_3_(aq)+ 5H_2_O(aq) (5)

Mg_3_Si_2_O_5_∙(OH)_4_ (s) + 6NH_4_HSO_4_ (aq)→ 3MgSO_4_ (aq) + 2SiO_2_ (s) + 5H_2_O (aq)+ 3(NH_4_)_2_SO_4_(aq) (6)

indicating that AS is formed when magnesium is extracted using ABS; Equation (6). The concentration of AS was hence also chosen to be a bit higher than ABS in the synthetic solutions prepared. It was also assumed that mainly ABS will contribute to the extraction of magnesium, which in this case meant that 0.16 M MgSO_4_ in the feed would react with ABS, thus resulting in the concentration of ABS of 0.54 M. Some magnesium will, however, also be extracted by AS. This amount will be smaller, however. [9,10,11] studied the effect of using AS versus ABS as leaching chemical, and found ABS to be much more effective.

**Figure 10:**
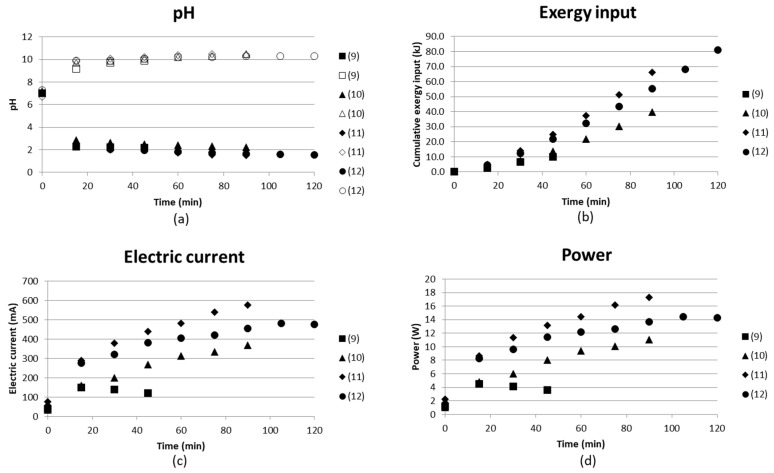
(**a**) pH in acidic and basic solution; (**b**) cumulative exergy input in kJ; (**c**) electric current in mA; and (**d**) power (W) as function of time for four continuous tests with decreasing flow rates from 1.14 L/h (9) to ≤2.5 L/h (12).

Figure 10 gives the pH in the compartments producing acidic solution and basic solution, the cumulative exergy input, electric current and the power as function of time. pH reached values of slightly above 10 and approximately 2 for the two products independent of flow rate. The flow rate decreases with increasing test number; see specific flow rates in Table 2. The electric current, however, varies more and the higher flow rates result in a lower current, and thus also lower exergy input. This could be partly explained by more ions having time to be separated at low flow rate. Test (12) used the concentrate outlet from (10) as starting solution, which did not seem to exhibit any effect of electric current or exergy input. Test (12) gave a result similar to the other tests ((9)–(11)), even if the feed had already been run through the BPMED once and the flow rates were kept low. The results could have been more easy to interpret if the flow rates had been over a wider range.

The conductivity of the ammonium-containing end solutions was measured and found to be low. The conductivity is still somewhat increased when the flow rate is lowered, see Table 5. pH in these solutions varied from 9.9 to 10.4, which indicates that any ammonium separated is converted to ammonia (NH_3_). The conductivity is affected by ions. The conductivity measured in the ammonia solutions might therefore be low, since dissolved molecular NH_3_ lacks the charge of an ion.

The following calculations give the total amount of NH_3_ + NH_4_^+^ in the basic solution of test (11):

[NH_4_^+^] = 0.3 g/L at pH 10.4 (Supplementary Material), K_b_ = 1.8 × 10^−5^

→ 93.3% is NH_3_ and 6.7% is NH_4_^+^ in B_out_ in test (11)

The total concentration is 0.233 mol(NH_3_)/L + 0.017 mol(NH_4_^+^)/L = 0.25 mol/L ≈ 4.5 g/L in B_out_, assuming that the molar mass is 18 g/mol, i.e., that of NH_4_^+^.

Similar to Section 3.1.1, an estimation of the energy requirement for separating one kg of NH_4_OH can be made:

→ [NH_4_^+^] = 4.5 g/L in 0.5L B_out_ requires 68 kJ of exergy (Figure 10)

→Separation 2.25 g [NH_4_^+^]/68 kJ

→ 302 MJ/kg NH_4_^+^ separated

#### 3.1.4. BPMED with Rock-Derived Solutions (Tests (13)–(15))

**Figure 11:**
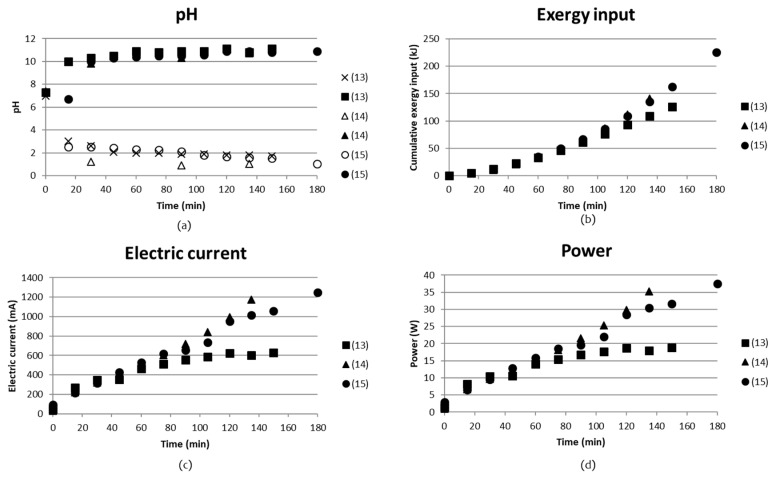
(**a**) pH; (**b**) cumulative exergy input; (**c**) electric current; and (**d**) power as function of time for tests run with rock-derived solutions.

Three tests ((13)–(15) in Table 2) were done using rock-derived solutions. Test (13) and (15) were run directly on rock-derived solutions, while test (14) used part of the concentrate outlet, C_out_, which was obtained after separation of acid and base in test (13). pH, cumulative exergy input, electric current and power as function of time are presented in Figure 11.

Analyses for SO_4_^2−^, NH_4_^+^, Mg^2+^, Fe^2+/3+^ and Ni^+/2+^ concentrations together with pH are shown in Table 6. Test parameters for the test are given in Table 2 and Table 3 above. It became clear that tests (13) and (15), where recirculation and continuous mode was applied, resulted in a very low separation degree. Comparing *C*_in_ with *C*_out_ for these tests showed that the concentration remained the same. The volume changes were not corrected for, which could possibly have changed these concentrations to some extent. On the other hand, even in the case where batch mode is applied, there was no remarkable volume change corrected for. This would have been visible even at minor volume changes since the compartments were full (98%) from the start and would overflow at significant volume changes. One important finding from these tests was the presence of Mg, Fe and Ni in the concentrate solution before and after the BPMED step. It seemed like the concentrations remained unchanged, which could indicate that membrane fouling did not become an issue. The presence of Mg^2+^ and Ca^2+^ may cause complex formation with hydroxides and in turn lead to fouling of the CMS membrane according to [29,30,31].

It seems as if a separation of SO_4_^2−^ of 10.8% was achieved in test (14), where the acidic solution in compartment (A) contained a solution of 14 g/L SO_4_^2−^ while the concentrate of the inlet solution decreased from 130 g/L to 110 g/L. However, a similar increase of SO_4_^2−^ was seen for test (15), with the difference that the analyzed SO_4_^2−^ in the concentrate solution (C) remained the same during the test. According to the external analyses report (Supplementary Material), the default difference was up to 10% in concentrates > 4 mg/L, which could explain the findings. The change from continuous flow in compartments A, B and C in test (13) to a recirculated flow in compartments A and B in test (15), seemed to improve the separation, since SO_4_^2−^ clearly was found in compartment A after the test.

The amount of NH_4_^+^ in compartment B in test (15) had increased by 9 times from 0.43 g/L to 3.9 g/L, compared to test (13). At pH above 11.5, however, it is present 100% as NH_3_ (molecular) in solution, while under pH 6.5 it is present as 100% NH_4_^+^ [32].

Calculations similar to Section 3.1.2 give the total amount of NH_3_ + NH_4_^+^ in the solution:

[NH_4_^+^] = 3.9 g/L at pH 10.9 (Table 6), K_b_ = 1.8 × 10^−5^

→ 97.8% is NH_3_ and 2.2% is NH_4_^+^ in B_out_ in test (15)

Total concentration is 9.71 mol(NH_3_)/L + 0.22 mol(NH_4_^+^)/L = 9.93 mol/L ≈ 178.74 g/L in B_out_, assuming that the molar mass is 18 g/mol, i.e., that of NH_4_^+^.

An estimation of the energy requirement for separating one kg of NH_4_OH can be made:

→[NH_4_^+^] = 178.74 g/L in 0.2L B_out_ (Table 6) requires 220 kJ of exergy (Figure 11)

→Separation 35.7g [NH_4_^+^]/220 kJ

→3.4 MJ/kg NH_4_^+^ separated

This energy demand is already almost 100 times lower than the previous energy/kg NH_4_^+^ values that were calculated in Section 3.1.1 and Section 3.1.1.

The solution from compartment 1 was used in test (13) in order to precipitate iron, but was not acidic enough to reach pH 8.5, which should be enough to precipitate both ferrous (II) and ferric (III) iron from the solution. The last test in Table 6 gives the concentrations of SO_4_^2−^, Mg, Fe and Ni in a solution obtained after the A_out_ from test (14) was used for leaching magnesium from Hitura rock. For this, 2 g of Hitura rock with particle size 63–125 µm was mixed with 75 mL of A_out_ at 70 °C in 3 h and insolubles were filtered. 25% of the rock was dissolved, which could be compared to 32.5 and 36%-wt dissolved in the leaching step preceding test (13) and (15).

Solution B_out_ in test (13) and (15) was used in order to increase pH in solution C_out_ in test (13) and (15). pH increased from 1.6 to 2.2, taking 100 mL of C_out_ and adding 415 mL of B_out_, so no iron was precipitated. A pH of 3.5 is needed in order to precipitate Fe(III) ions, and pH 8.5 is enough to precipitate Fe(II). When the operation was changed to recirculation of the acidic and basic solution, it was possible to precipitate 0.3 g iron product by taking 50 mL of C_out_ and 94 mL of B_out_, which was enough to increase pH from 1.6 to 8.5. Since very small amounts of B_out_ were left after this, it was not possible to run a carbonation test, i.e., add B_out_ until pH 10 was reached and then insert CO_2_ gas. Adjustments needs still to be done in order to reach more acidic and basic solutions from the BPMED.

Total concentration of sulfate was almost the same as sulfate and bisulfate combined as 0.7 M AS and 0.7 M ABS. The analyses only took SO_4_^2−^ ion into account, which in this case means that practically all bisulfate ions had converted to sulfate ions during the leaching of the rock. This is an important point to take into account when designing the BPMED setup and operation, since bisulfate is a monovalent ion, while sulfate is a divalent ion. 0.7 M AS contributes to 0.7 M SO_4_^2−^ and 0.7 M ABS contributes also with a maximum of 0.7 M SO_4_^2−^ if all HSO_4_^2−^ ions give off a H^+^ ion. The maximum concentration of SO_4_^2−^ is thus 1.4 M, which corresponds to a concentration of 1.4 (mol/L)∙96.06 (g/mol) = 134.5 g/L. The concentration of analyzed SO_4_^2−^ in *C*_in_ was 130 g/L, which is 96.6% of the calculated value in Equation (5). Another explanation of the high concentration of SO_4_^2−^ could be that SO_4_^2−^ from the electrode compartments is being permeating through the BPM, although according to [33], the cations permeate easier through the BPM than anions.

### 3.2. BPMED after Carbonation

**Figure 12:**
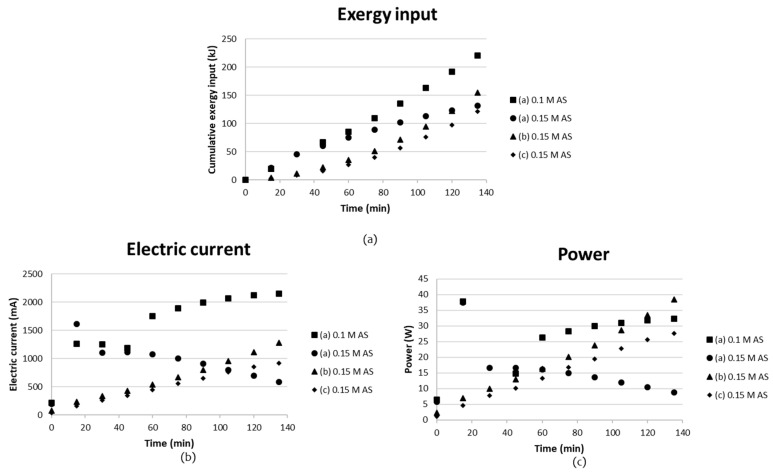
(**a**) cumulative exergy input; (**b**) electric current; and (**c**) power as a function of time for tests with three different BPMED setups aiming at separation of AS and/or ABS from ammonia after the carbonation step.

BPMED tests with three different setups were tested briefly in batch mode. These are referred to as setups (a)–(c), respectively, in Figure 6. The volume changes as a result of water transport through the BPMs was not taken into account, since all compartments were filled with solutions and there were no significant volume changes. Electric current, power and the cumulative exergy input for the tests are given in Figure 12. The current increased rapidly in the tests with setup (a) (Figure 6), resulting in a decreasing voltage. To keep the voltage constant as for the other tests, the voltage was adjusted to 15 V after 15 and 30 min, respectively. The cationic membrane changed color during both tests, indicating that fouling might have occurred. This might have caused the voltage drop. 15 V was chosen, since it seemed to be a suitable level at which the voltage could be kept constant without limiting the current going through the stack. The reason for the voltage drop should be further investigated. The electric current increased with time in setup (a) when 1.0 M AS + ammonia was used, but this decreased as the concentration was lowered to 0.15 M AS + ammonia. This might also indicate some weaknesses of the chosen setup (a).

Setup (a) (Table 4) included one of the BPMs arranged so that the anionic side of the membrane faced the cathode compartment. Placing the cationic side towards the cathode will result in highly proton selective membrane as a result of a high dynamic resistance [23]. In the case of setup (a), this dynamic resistance was inhibited, which might have resulted in the unstable performance. The BPM could not separate any ions, since it was placed in such a way that the electric current as a result counteracted separation.

Table 7 presents the distribution of sulfate and ammonia between the compartments before (in) and after (out) the test. The analyses are made of the entire solutions collected from the individual compartments after the test.

Clearly, a separation took place in both the high and low concentration tests using setup (a), but SO_4_^2−^ and NH_4_^+^ were found to quite a high extent in almost all compartments. The test with setup (a) must be further investigated with respect to the membranes, since the voltage drop and the color change of the CMS membranes indicated that setup (a) did not work properly. The transport of ammonium ions should primarily take place between compartments 1 and 2, but hardly any ammonium was transported from compartment 1. Setup (a) clearly inhibited the transport of ammonium in this direction. Many highly mobile H^+^ ions are available at low pH and will be able to carry the current instead of NH_4_^+^.

According to the distribution calculations, a separation of 40% of NH_4_^+^ using setup (b) was achieved. A separation degree of 42% NH_4_^+^ from compartment 2 to compartment 3 was achieved in setup (c). The intention with compartment 1 in (c) was mainly to let OH^−^ from compartment 2 and H^+^ from the BPM be combined with water to maintain charge balance. Looking at the pH of compartment 1 with setup (c), however, it seems as if there was a surplus of H^+^ and that sulfate ions have been transported to equalize the positively charged ions. In setup (b), on the other hand, only four compartments were used, and thus no energy was needed for “water combination”. Running more tests using setup (b) or (c) would be valuable for achieving a deeper understanding and further optimization.

Calculations similar to Section 3.1.2. and Section 3.1.3 give the energy need for separating 1 kg of NH_4_^+^ using setup (c);

[NH_4_^+^] = 5.2 g/L at pH 11.2 (Supplementary Material, Table 7), K_b_ = 1.8 × 10^−5^

→ 98.9% is NH_3_ and 1.1% is NH_4_^+^ in Comp. 3 in setup (c)

Total concentration is 28.9 mol(NH_3_)/L + 0.33 mol(NH_4_^+^)/L = 29.2 mol/L ≈ 525.6 g/L in Comp. 3 (c), assuming that the molar mass is 18 g/mol, i.e., that of NH_4_^+^.

An estimation of the energy requirement for separating one kg of NH_4_OH can be made:

→ [NH_4_^+^] = 525.6 g/L in 0.135 L Comp. 3 requires 120 kJ of exergy (Figure 12)

→Separation 71.0 g [NH_4_^+^]/120 kJ

→ 1.7 MJ/kg NH_4_^+^ separated

Four different tests, (4), (11), (15) and (c), respectively, were then studied, with a focus on the separation of ammonium ions from the process stream versus energy demand for the separation. A summary of these results is presented in Table 8 below. Test (4) was the only test in Table 8 where NH_4_^+^ was measured in the concentrate having pH well below 6.5, i.e., 100% of NH_4_^+^ ions in solution. NH_4_^+^ ions were also measured by ISE. Samples from the other three tests were sent for external analysis, with pH well above 6.5. These were therefore recalculated to take dissolved molecular NH_3_ into account. Looking at the energy demand for the separation of 1 kg from the process stream, there seems to be a lowest energy demand of 1.7 MJ/kg NH_4_^+^ in the case of setup (c) after carbonation was applied. The energy for the separation was approximately 200 times less than in the worst case of the four, i.e., test (4) when a synthetic low concentration solution was used. From these calculations, it would be interesting to further study the application of BPMED after carbonation, here (c), in order find and develop options that are more energy effective.

## 4. Conclusions

Different setups using BPMED were used in this study, with the aim of finding ways to recover chemicals used for mineral carbonation. These tests with the aim of separating AS and/or ABS from ammonia from the process solution after the carbonation step seem to have potential. The combination of two different ED steps could make the regeneration and use of especially ammonia still more efficient. However, the design and operation of the setup must be investigated further in order to optimize it and to make the separations more energy effective. One option is to run tests with a commercial laboratory-scale BPMED setup, with a narrower compartment width and more repeating units than was used here.

External analysis reports showed that iron, magnesium and nickel will pass the ED stack without causing any serious fouling. The fouling in used membranes should, however, be studied in order to confirm the analysis results.

BPMED tests with rock-derived solutions after carbonation should be an important part of further developing a suitable mineral carbonation route including efficient regeneration of chemicals involved. Rock-derived solutions, however, still require improved separation of ammonia after the leaching step to be able to use the ammonia solution for the precipitation of iron as well as the carbonation step. Rock-derived solutions were, for this reason, not used here for the BPMED separation tests after carbonation. Some attempts were made to use recovered basic solution from the BPMED to first precipitate iron and then carbonate the magnesium. The amount of basic solution, however, was only sufficient to precipitate iron, and at this time, carbonation could not be accomplished by regeneration of the starting chemicals.

Comparison of the energy demands for separating 1 kg of NH_4_^+^ between four tests were also made. The energy, i.e., input electricity needed, was ranging from 1.7 and 3.4 MJ/kg NH_4_^+^ to 302 and up to 350 MJ/kg NH_4_^+^ in tests (c), (15), (11) and (4), respectively. From this point of view, it would be interesting to further study the operation and the design of the setups used in (15) and (c), respectively. Test (15) used solutions after leaching for separation, while (c) focused on separation of ammonium from solutions after carbonation. This is a positive outcome, and more focus could still be placed on applying ED both after leaching and after carbonation, at least looking at the lower energy demands resulting from both arrangements of the membranes.

This study has shown that it is possible to separate ammonium and sulfates for the regeneration of chemicals by BPMED and this should be part of fully developing the ÅA route to a process with low energy regeneration of flux salts needed for CO_2_ sequestration through mineral carbonation.

## Figures and Tables

**Table 1:** Composition of the rock used for tests. 70–80%-wt of the rock consists of serpentinite, 15–20%-wt of magnetite (Fe_3_O_4_) and <5% of chlorite and calcite, respectively.

Compound	%-wt, Dry
CaO	0.4
SiO_2_	35.5
TiO_2_	0.04
NiO	0.35
Al_2_O_3_	0.45
Fe_2_O_3_	14.6
MgO	35.1
K_2_O	0.02
Na_2_O	<0.01
MnO	0.12
CuO	0.10
Cr_2_O_3_	0.38
P_2_O_5_	0.01
S-Eltra	0.42
LOI 1000 °C	12.0
Rest	0.5

**Table 2:** Process parameters for ED tests with synthetic and rock-derived solutions from the leaching step. The *-marked tests are tests where the BPM was replaced by a CMS and an ACS membrane placed together. These test will not be addressed in any graphs in the results section.

Test	Synthetic or Rock-Derived	Rinse Solution	Voltage (V)	Total Time (min)	Mode (Recirc./Batch/Cont.) Flow Rate Conc. Compartment	Mode (Recirc./Batch/Cont.) Flow Rate Acid + Base Compartments
(1) *	Synthetic	H_2_O	20	135	Batch	Batch
(2) *	Synthetic	H_2_O	30	135	Batch	Batch
(3)	Synthetic	H_2_O	20	135	Batch	Batch
(4)	Synthetic	0.05/0.05	20	135	Batch	Batch
(5)	Synthetic	AS/ABS 0.05/0.05	20	135	Batch	Batch
(6)	Synthetic	AS/ABS 0.05/0.05	20	90	Recirc. 1 L/h	Recirc. 1 L/h
(7)	Synthetic	AS/ABS 0.05/0.05	20	90	Recirc. 1 L/h	Recirc. 1L/h
(8)	Synthetic	AS/ABS 0.05/0.05	20	105	Cont. 1 L/h	Recirc. 1 L/h
(9)	Synthetic	AS/ABS 0.05/0.05	30	53	Cont. 1.14 L/h	Cont. 1.14 L/h
(10)	Synthetic	AS/ABS 0.05/0.05	30	120	Cont. 0.56 L/h	Cont. 0.64 + 0.62 L/h
(11)	Synthetic	AS/ABS 0.05/0.05	30	90	Cont. 0.67 L/h	Cont. 0.33 L/h
(12)	Synthetic	AS/ABS 0.05/0.05	30	120	Cont. 0.25 L/h	Cont. 0.2 L/h
(13)	Rock der.	AS/ABS 0.05/0.05	30	143	Cont. 0.25 L/h	Cont. 0.275 L/h
(14)	Rock der.	0.15 M AS	30	135	Batch	Batch
(15)	Rock der.	0.15 M AS	30	180	Cont. 0.25 L/h	Recirc. 0.084 L/h

**Table 3:** Composition of starting solutions or leaching conditions for tests given in Table 2.

	Composition of Starting Solution				
Test	ABS	AS	MgSO_4_	Fe(II)SO_4_∙7H_2_O	Fe(III)_2_(SO_4_)_3_∙H_2_O
(1)	0.05 M	0.05M	-	-	-
(2)	0.05 M	0.05 M	-	-	-
(3)	0.05 M	0.05 M	-	-	-
(4)	0.05 M	0.05 M	-	-	-
(5)	0.05 M	0.05 M	0.03 M	0.01 M	-
(6)	0.05 M	0.05 M	0.03 M	0.01 M	-
(7)	0.05 M	0.05 M	0.03 M	0.01 M	-
(8)	0.05 M	0.05 M	0.03 M	0.01 M	-
(9)	0.54 M	0.7 M	0.16 M	0.009 M	0.0015 M
(10)	0.54 M	0.7 M	0.16 M	0.009 M	0.0015 M
(11)	0.54 M	0.7 M	0.16 M	0.009 M	0.0015 M
(12)	C(out) from test (10)				
(13)	Solution from leaching: Hitura, 63–125 µm, 70 °C, 0.8 L, 20 g				
(14)	C(out) from test (13)				
(15)	Solution from leaching: Hitura, 63–125 µm, 70 °C, 0.8 L, 20 g				

**Table 4:** Process parameters for ED tests with synthetic solutions prepared to match the solution composition after the carbonation step. * Ammonia solution was added to the initial AS solution to get a solution with pH 10. “+” and “–“ refer to the anode and the cathode, respectively.

Test	Setup (Figure 6)	Number of Compartments	Initial AS Solution *	Membrane Order
1	(a)	5	1.0 M	+BPM(ACS/CMS)-CMS-ACS-BPM(CMS/ACS)-
2	(a)	5	0.15 M	+BPM(ACS/CMS)-CMS-ACS-BPM(CMS/ACS)-
3	(b)	4	0.15 M	+BPM(CMS/ACS)-CMS-BPM(ACS/CMS)-
4	(c)	5	0.15 M	+BPM(ACS/CMS)-ACS-CMS-BPM(ACS/CMS)-

**Table 5:** Conductivities and pH in end basic solutions for tests run at different flow rates. Test (12) used concentrate outlet solution from (10) as starting solution.

Test Number	(9)	(10)	(11)	(12)	H_2_O _in_
Conductivity_stop_ (µS/cm)	70	140	420	295	30
pH	9.9	10.4	10.4	10.3	7

**Table 6:** Concentrations of SO_4_^2−^, NH_4_^+^, Mg^2+^, Fe^2+/3+^ and Ni^+/2+^ in the starting solution going into the BPMED stack (C in), and in the solution obtained after the test (A, B and C out, respectively). Comp. = compartment; A = acidic solution, B = basic solution, C = concentrate. Test (14) started with solution C out from test (13).

Test	Sample Type	Comp. No.	Vol. Tot. (L)	SO_4_^2−^ (g/L)	NH_4_^+^ (g/L)	Mg^2+^ (g/L)	Fe^2+/3+^ (g/L)	Ni^+/2+^ (g/L)	pH
13	C_in_	Comp. 2		130		2.9	0.69	0.027	1.8
13	A_out_	Comp. 1	0.8	2.3					1.7
13	C_out_	Comp. 2	0.75	130	37	2.8	0.68	0.027	1.8
13	B_out_	Comp. 3	0.8		0.43				11.1
14	C_in_ = C_out_(13)	Comp. 2		130		2.8	0.68	0.027	1.5
14	A_out_	Comp. 1	0.135	14					1.0
14	C_out_	Comp. 2	0.135	110	31	2.7	0.66	0.026	1.8
14	B_out_	Comp. 3	0.135		1.7				10.7
15	C_in_	Comp. 2		130		2.7	0.64	0.026	1.9
15	A_out_	Comp. 1	0.2	15					1.0
15	C_out_	Comp. 2	0.75	130	35	2.7	0.66	0.026	1.7
15	B_out_	Comp. 3	0.2		3.9				10.9
	C_L2_			14		2.2	0.29	0.021	2.3

**Table 7:** Distribution of total amount of sulfate and ammonium ions in the compartments (= Comp) before any electric current is applied (in) and after the test (out). Setups (a), (b) and (c) are presented in Figure 6.

Setup	Concentration in (M)	Compartment	SO_4_^2−^ in (%)	NH_4_^+^ in (%)	SO_4_^2−^ out (%)	NH_4_^+^ out (%)	pH out
(a)	1.0 AS+ NH_4_OH to pH 10	Comp. 1	50	50	46.0	46.3	9.2
(a)	Dest. H_2_O	Comp. 2 NH_4_OH	0	0	14.9	16.5	9.7
(a)	1.0 AS+ NH_4_OH to pH 10	Comp. 3 AS	50	50	39.1	37.2	9.6
		SUM	100	100	100	100	
(a)	0.15 AS+ NH_4_OH to pH 10	Comp. 1	50	50	51.6	35.3	8.9
(a)	Dest. H_2_O	Comp. 2 NH_4_OH	0	0	45.2	47.1	9.4
(a)	0.15 AS+ NH_4_OH to pH 10	Comp. 3	50	50	3.2	17.6	10.4
		SUM	100	100	100	100	
(b)	0.15 AS+ NH_4_OH to pH 10	Comp. 1	100	100	99.2	60.0	9.8
(b)	Dest. H_2_O	Comp. 2 NH_4_OH	0	0	0.8	40.0	11.3
		SUM	100	100	100	100	
(c)	Dest. H_2_O	Comp. 1	0	0	76.6	6.5	1.2
(c)	0.15 AS+ NH_4_OH to pH 10	Comp. 2	100	100	23.0	51.6	10.2
(c)	Dest. H_2_O	Comp. 3 NH_4_OH	0	0	0.4	41.9	11.2
		SUM	100	100	100	100	

**Table 8:** Energy requirement for separating 1 kg of NH_4_^+^ in four different types of tests.

Test	Total Volume (L)	Total Time (min)	pH in Solution Analyzed	Analysis Method	Analysis from Sol.	Synthetic or Rock-Derived	MJ/kg NH_4_^+^
(4)	0.135	135	2.2	ISE	Conc. sol.	Synth.	350
(11)	0.5	90	10.4	External	Basic sol.	Rock	302
(15)	0.2	180	10.9	External	Basic sol.	Synth.	3.4
(c)	0.135	135	11.2	External	Basic sol.	Synth.	1.7

## Supplementary Materials

The following are available online at https://www.mdpi.com/1099-4300/21/4/395/s1, Document S1: Analysis report part I and part II.

## Abbreviations

ÅA	Åbo Akademi University
A	acidic solution (in Figure 3, Figure 5 and Figure 6 indicating also anion membrane)
ABS	ammonium bisulfate, NH_4_HSO_4_
ACS	monovalent anion exchange membrane
AS	ammonium sulfate, (NH_4_)_2_SO_4_
B	basic solution
BPM	bipolar membrane
BPMED	bipolar membrane electrodialysis
C	concentrate (in Figure 3, Figure 5 and Figure 6 indicating also cation membrane)
CMS	monovalent cation exchange membrane
ED	electrodialysis
ISE	ion selective electrode
