# Supplementary material for: Energy Use of Flux Salt Recovery Using Bipolar Membrane Electrodialysis for a CO2 Mineralisation Process
Source: Entropy (Basel). 2019 Apr 12;21(4):395. doi: 10.3390/e21040395 (PMC7514884; doi:10.3390/e21040395)
Supplement: Supplementary file 1 [file entropy-21-00395-s001.pdf]

SUPPLEMENTARY MATERIAL S1: Analysis results part I and part II

eurofins

Tutkimustodistus **AR-19-RZ-001240-01** **Sivu 1/3**
Päivämäärä **14.01.2019**

Åbo Akademi, water samples

**Näytenumero**			**750-2018-00026398**	**750-2018-00026399**	**750-2018-00026400**	**750-2018-00026401**	**750-2018-00026402**
**Näytteen nimi**			A 28.11.18	B 28.11.18	C 28.11.18	A 13.12.18	B 13.12.18
**Näytteen kuvaus**			Muut nestemäiset materiaalit	Muut nestemäiset materiaalit	Muut nestemäiset materiaalit	Muut nestemäiset materiaalit	Muut nestemäiset materiaalit
**Yleiset vedestä tehtävät tutkimukset**							
Ammonium (NH4)	RZC29	mg/l		300	35000		430
Sulfaatti (SO4)	RZB86	mg/l	1500		130000	2300	
**Alkuaineet, kokonaispitoisuus, HNO3, ICP-MS**							
Magnesium (Mg)	RZ0IR	mg/l			2100		
Nikkeli (Ni)	RZ0IB	mg/l			0,077		
Rauta (Fe)	RZ0IG	mg/l			540		
Mikroaaltohajotus	RZE24				Tehty		

**Näytenumero**			**750-2018-00026403**	**750-2018-00026404**	**750-2018-00026405**	**750-2018-00026406**	**750-2018-00026407**
**Näytteen nimi**			C 13.12.18	A 19.12.18	B 19.12.18	C 19.12.18	A 20.12.18
**Näytteen kuvaus**			Muut nestemäiset materiaalit	Muut nestemäiset materiaalit	Water	Water	Water
**Yleiset vedestä tehtävät tutkimukset**							
Ammonium (NH4)	RZC29	mg/l	37000		1700	31000	
Sulfaatti (SO4)	RZB86	mg/l	130000	14000		110000	15000
**Alkuaineet, kokonaispitoisuus, HNO3, ICP-MS**							
Magnesium (Mg)	RZ0IR	mg/l	2800			2700	
Nikkeli (Ni)	RZ0IB	mg/l	27			26	
Rauta (Fe)	RZ0IG	mg/l	680			660	
Mikroaaltohajotus	RZE24		Tehty			Tehty	

**Näytenumero**			**750-2018-00026408**	**750-2018-00026409**	**750-2018-00026410**	**750-2018-00026411**	**750-2018-00026412**
**Näytteen nimi**			B 20.12.18	C 20.12.18	Mg-rich 11.12.18	Mg-rich 19.12.18	Mg-rich L2 20.12.18
**Näytteen kuvaus**			Water	Water	Water	Water	Water
**Yleiset vedestä tehtävät tutkimukset**							
Ammonium (NH4)	RZC29	mg/l	3900	35000			
Sulfaatti (SO4)	RZB86	mg/l		130000	130000	130000	14000
**Alkuaineet, kokonaispitoisuus, HNO3, ICP-MS**							

eurofins

Tutkimustodistus AR-19-RZ-001240-01 **Sivu 2/3**
Päivämäärä 14.01.2019

**Näytenumero**			**750-2018-00026408**	**750-2018-00026409**	**750-2018-00026410**	**750-2018-00026411**	**750-2018-00026412**
**Näytteen nimi**			B 20.12.18	C 20.12.18	Mg-rich 11.12.18	Mg-rich 19.12.18	Mg-rich L2 20.12.18
**Näytteen kuvaus**			Water	Water	Water	Water	Water
Magnesium (Mg)	RZ0IR	mg/l		2700	2900	2700	2200
Nikkeli (Ni)	RZ0IB	mg/l		26	27	26	21
Rauta (Fe)	RZ0IG	mg/l		660	690	640	290
Mikroaaltohajotus	RZE24			Tehty	Tehty	Tehty	Tehty

eurofins

Tutkimustodistus AR-19-RZ-001240-01 Sivu 3/3
Päivämäärä 14.01.2019

Menetelmätiedot

Testikoodi	Parametrin nimi	Menetelmän mittausepävarmuus	Menetelmän määritysraja	Akkreditoitu	Menetelmä	Laboratorio
**Yleiset vedestä tehtävät tutkimukset**						
RZC29	Ammonium (NH4)	20%(<13mg/l) 10%(=13mg/l)	2	Kyllä	Sis. men. EF2034, Kjeldahl (titraus)	RZ T039
RZB86	Sulfaatti (SO4)	12%(<4mg/l) 10%(>4mg/l)	0.5	Kyllä	Sis. men. EF2018, IC-EC	RZ T039
**Alkuaineet, kokonaispitoisuus, HNO3, ICP-MS**						
RZ0IR	Magnesium (Mg)	20%	0.1	Kyllä	SFS-EN ISO 17294-2	RZ T039
RZ0IB	Nikkeli (Ni)	20%	0.003	Kyllä	SFS-EN ISO 17294-2	RZ T039
RZ0IG	Rauta (Fe)	20%	0.025	Kyllä	SFS-EN ISO 17294-2	RZ T039
RZE24	Mikroaaltohajotus			Kyllä	SFS-EN ISO 15587-2	RZ T039

Laboratorio

RZ T039	Eurofins Environment Testing Finland (Lahti)	FINAS akkr. num. SFS-EN ISO/IEC 17025:2005 FINAS T039

Menetelmäkuvaukset

SFS-EN ISO 15587-2
SFS-EN ISO 17294-2
Sis. men. EF2018
Sis. men. EF2034

eurofins

Analytical Report Nr. **AR-19-RZ-003618-02** **Page 1/2**
Date **18.02.2019**

Åbo Akademi, water samples

**Sample number**			**750-2019-00003728**	**750-2019-00003729**	**750-2019-00003730**	**750-2019-00003731**	**750-2019-00003732**
**Sample reference**			Conc in 17.1.2019	Cell 1 17.1.2019	Cell 2 NH4OH 17.1.2019	Cell 3 17.1.2019	Cell 1 18.1.2019
**Sample description**			Water	Water	Water	Water	Water
**General analyses of water**							
Sulphate (SO4)	RZB86	mg/l	85000	99000	32000	84000	16000
Ammonium (NH4)	RZC29	mg/l	60000	56000	20000	45000	6600

**Sample number**			**750-2019-00003733**	**750-2019-00003734**	**750-2019-00003735**	**750-2019-00003736**	**750-2019-00003737**
**Sample reference**			Cell 2 18.1.2019	Cell 3 18.1.2019	Cell 1 22.1.2019	Cell 2 22.1.2019	Cell 1 "H2O" 23.1.2019
**Sample description**			Water	Water	Water	Water	Water
**General analyses of water**							
Sulphate (SO4)	RZB86	mg/l	14000	1000	7800	63	10000
Ammonium (NH4)	RZC29	mg/l	8800	3300	6300	4200	800

**Sample number**			**750-2019-00003738**	**750-2019-00003739**
**Sample reference**			Cell 2 conc 23.1.2019	Cell 3 NH4OH 23.1.2019
**Sample description**			Water	Water
**General analyses of water**				
Sulphate (SO4)	RZB86	mg/l	3000	56
Ammonium (NH4)	RZC29	mg/l	6400	5200

eurofins

Analytical Report Nr. AR-19-RZ-003618-02 Page 2/2
Date 18.02.2019

Method information

Testcode	Parameter name	Default MU	Default LOQ	Accredited	Method	Laboratory
**General analyses of water**						
RZB86	Sulphate (SO4)	12%(<4mg/l)<br>10%(>4mg/l)	0.5	Yes	Int. Method, IC, based on e.g. SFS-EN ISO 10304-1:2009, IC-EC	RZ T039
RZC29	Ammonium (NH4)	20%(<13mg/l)<br>10%(=13mg/l)	2	Yes	SFS 5505:1988 mod.	RZ T039

Laboratory

RZ T039	Eurofins Environment Testing Finland (Lahti)	FINAS acc num. SFS-EN ISO/IEC 17025:2005 FINAS T039

Method
Int. Method, IC, based on e.g.<br>SFS-EN ISO 10304-1:<br>SFS 5505:1988 mod.
